# Fatigue in Women With Gynecologic Cancer Compared to Patients With Other Cancers Receiving Chemotherapy

**DOI:** 10.1093/geroni/igaf122.3202

**Published:** 2025-12-31

**Authors:** David Asakitogum, Arsham Alamian, Victoria Loerzel

**Affiliations:** University of Central Florida, Orlando, Florida, United States; University of Miami, Coral Gables, Florida, United States; University of Central Florida, Orlando, Florida, United States

## Abstract

Cancer-related fatigue is reported as the most worrisome symptom in people with cancer. Evidence suggests that chemotherapy is associated with higher levels of fatigue and fatigue variability exists in patients with different cancers. However, there is limited research that compare fatigue levels in patients with gynecologic cancer and other cancers. Therefore, this study aimed to: evaluate and compare fatigue levels in patients with gynecologic, breast, colo-rectal, head and neck cancers; and evaluate demographic and experiential characteristics associated with fatigue severity in these cancer types. Older outpatients (≥50 years) with cancer (n = 99) were recruited before initiation of chemotherapy. Patients completed the Symptom Representation Questionnaire and the EORTC-Core 30 over four cycles of chemotherapy. Parametric and non-parametric tests were used to evaluate differences within and between groups. Logistic regression was used to identify risk factors associated with the severity of fatigue. Patients with gynecologic cancer reported higher mean fatigue scores at Baseline and at Time-4 [3.18(2.63) and 4.81(3.03), respectively] compared to other cancers. Patients with breast cancer reported the highest increased of mean fatigue score from Baseline to Time-4 [2.25(2.54) Vs 4.33(3.38)] (i.e., 94.44%). Patients with gynecologic and breast cancer were more likely to report moderate or severe fatigue at baseline and Time-4 compared to other cancers [(OR = 2.68, 95% CI: 0.873 – 8.206, p = 0.085); OR = 4.90, 95% CI: 0.671 – 35.819, p = 0.117] respectively. Patients with gynecologic cancer experienced higher levels of fatigue compared to other patients with cancer. Clinicians can use this information to identify high risk patients for early fatigue interventions.

